# Translation, cultural adaptation and validation of the Brazilian version of the Alopecia Areata Quality of Life Index (AA-QLI-BRA)^☆^

**DOI:** 10.1016/j.abd.2025.501146

**Published:** 2025-06-28

**Authors:** Paula Rosa Coutinho Goulart Borges Mariottoni, Leonardo Spagnol Abraham, Daniel Fernandes Melo, Rodrigo Pirmez, Leopoldo Duailibe Nogueira Santos, Paulo Müller Ramos, Hélio Amante Miot

**Affiliations:** a Department of Infectology, Dermatology, Imaging Diagnosis and Radiotherapy, Faculty of Medicine, Universidade Estadual Paulista, Botucatu, SP, Brazil; b Trichology Outpatient Clinic, Hospital Regional da Asa Norte, Brasília, DF, Brazil; c Trichology Outpatient Clinic, Universidade do Estado do Rio de Janeiro, Rio de Janeiro, RJ, Brazil; d Hair Studies Center, Santa Casa da Misericórdia do Rio de Janeiro, Rio de Janeiro, RJ, Brazil; e Alopecia Outpatient Clinic, Santa Casa de São Paulo, São Paulo, SP, Brazil

*Dear Editor,*

Alopecia areata (AA) is a chronic autoimmune disease that affects up to 1% of the population during their lifetime, regardless of age, gender, or ethnicity. Severe forms are challenging, requiring treatments that include corticosteroids, immunomodulators, and, more recently, Janus Kinase (JAK) inhibitors, which represent a new hope for refractory cases.^1^, ^2^

The impact on the Quality of Life (QoL) of patients with AA goes beyond hair loss. Because it is a socially noticeable condition, it affects self-esteem and self-image and can lead to feelings of shame, isolation, and stigmatization. Therefore, many patients develop symptoms of anxiety and depression, especially in more severe cases. The psychological impact is more pronounced in children and adolescents due to their greater emotional vulnerability. Moreover, its unpredictable and recurrent nature contributes to a state of uncertainty, affecting daily activities, relationships, and work productivity.^3^, ^4^, ^5^

Fabroccini et al. developed a specific unidimensional instrument to investigate the impact on QoL in adults with AA, the Alopecia Areata Quality of Life Index (AA-QLI).^6^ This self-administered instrument consists of 21 items related to Symptoms (S), Relationship (R) and Objective (O) signs.

A methodological study was carried out to adapt and validate the Brazilian version of the AA-QLI. After authorization for the translation of the material (#2023-5634220334356), four dermatologists, fluent in English, translated it into Brazilian Portuguese, generating a consensual version (AA-QLI-BRA), which was back-translated into English by a non-specialist and compared to the original version. To obtain the cultural adaptation, ten patients with AA evaluated the instrument and were asked about the clarity of the questions, the language used, and its applicability. The version adapted to Brazilian Portuguese is available at https://doi.org/10.17632/jw2fxprgd3.1.

For content validation, seven dermatologists with experience in trichology evaluated and scored (from 0 to 10) each item for relevance, with 0 being – Not at all relevant and 10 being – Extremely relevant.

For the other validations, 80 adults with AA (diagnosed by a dermatologist) were investigated using an online questionnaire containing demographic data of AA-QLI-BRA and the DLQI-BRA (Dermatology Quality of Life Index), for concurrent validation. The study was conducted at the Trichosis outpatient clinic of FMB-UNESP, the Trichology outpatient clinic of Hospital Regional da Asa Norte (HRAN), the Trichology outpatient clinic of UERJ, Hair Studies Center of the Santa Casa da Misericórdia do Rio de Janeiro and the Alopecia outpatient clinic of Santa Casa de São Paulo.

A subgroup of nine participants completed the questionnaire again within a 14-day period to investigate its temporal stability. The instrument's internal consistency was assessed using the McDonald's coefficient. The “floor” and “ceiling” effects were defined when extreme responses (“not at all” or “too much”) accounted for more than 50% of the responses to an item.^7^

The participants’ demographic and QoL findings are shown in Table 1. There was a predominance of women over 30 years of age, with higher levels of schooling and extensive forms of AA. During the in-person application, the instrument was completed in less than ten minutes.

**Table 1:** Demographic, clinical and quality of life data of 80 patients with AA.

Variables		Values
Age (years)	Mean (SD)	36 (15)
Sex^a^	Female	60 (75%)
	Male	20 (25%)
Level of schooling^a^	Elementary School	7 (9%)
	High School	13 (16%)
	Higher Education	60 (75%)
Marital status^a^	Married	25 (31%)
	Divorced	8 (10%)
	Single	47 (59%)
Self-declared skin color^a^	White	49 (61%)
	Non-white	31 (39%)
Age at onset (years)	Mean (SD)	24 (15)
Extent of alopecia	Small areas	36 (45%)
	Total	19 (24%)
	Universal	25 (31%)
DLQI-BRA	Median (Q1‒Q3)	7 (1‒15)
AA-QLI-BRA	Mean (SD)	53 (17)

a n (%). SD, Standard Deviation; Q1‒Q3, First and third quartiles.

The scores for each item of the AA-QLI-BRA are shown in Fig. 1. Item S4 (worry that the problem will last for the rest of one’s life) showed a ceiling effect. The floor effect occurred in items R15 (people are afraid of contagion) and R17 (difficulty establishing relationships).

**Fig. 1:**
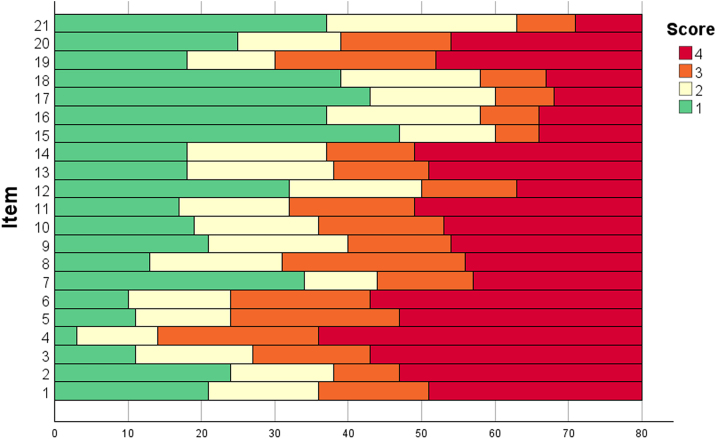
Distribution of item scores of patients with AA-QLI-BRA (n = 80).

Content validity was confirmed by the mean score of experts >8 for all the instrument items, except S1 (uncomfortable with a wig), S8 (expenses for hair care) and O21 (pruritus in the scalp).

The internal consistency of the AA-QLI-BRA was 0.95 (95% CI 0.94‒0.97) and of the DLQI was 0.93 (95% CI 0.91‒0.95). The correlation (Spearman's rho) between the AA-QLI-BRA and DLQI scores was 0.88 (p < 0.01). The correlation of the scores of the AA-QLI-BRA items among themselves and between them and the total (Fig. 2) indicated lower coefficients related to item O21.

**Fig. 2:**
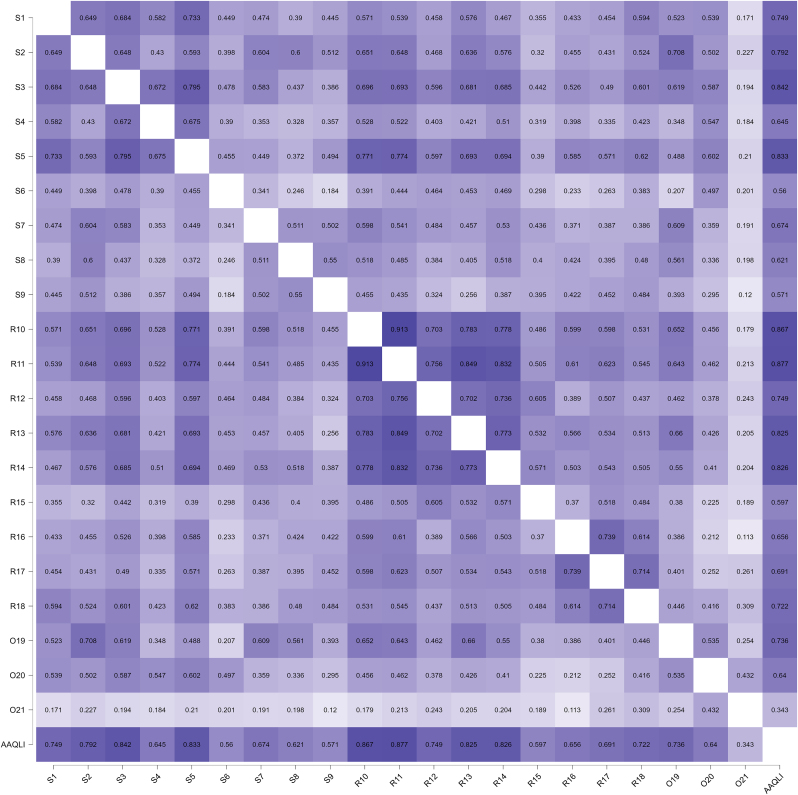
Heat map of correlations (Spearman's rho) between AA-QLI-BRA items and the total score (n = 80).

The exploratory factor analysis, using the principal axis factoring method, indicated that 49.0% of the construct variance was explained by the unidimensional factor. The KMO coefficient for the matrix was 0.91 and the sphericity test (Bartlett) was p < 0.001, indicating sample adequacy for analysis. All items showed a factor loading >0.45, except for O21, which was 0.33.^7^, ^8^, ^9^

The network analysis using the EBICglasso method (Fig. 3) showed greater influence for the AA-QLI-BRA items: R11 (people notice my problem) and S5 (I can't forget that I have the problem). Items S4, S6 (fear of the disease spreading), R15 and O21, on the other hand, showed less influence in the network.

**Fig. 3:**
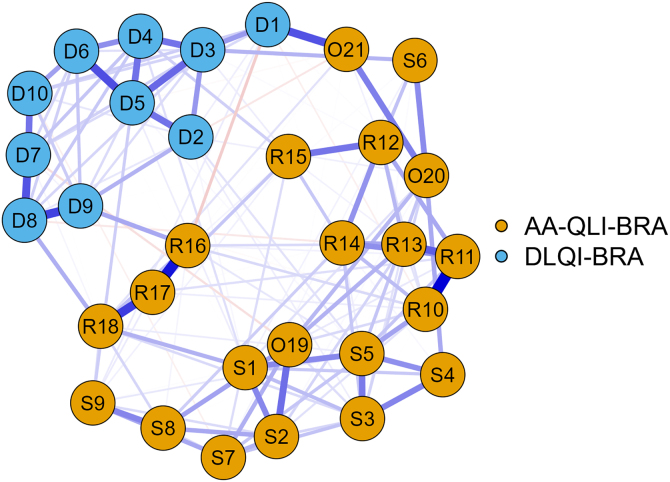
Network diagram between DLQI-BRA and AA-QLI-BRA items (n = 80).

The exclusion of items S4, S6, R15 and O21 did not change the instrument’s internal consistency, and the variance of the explained latent variable increased to 54.7%.

In the temporal stability assessment, the mean (standard deviation) of the test scores was 58 (11), and in the retests, 57 (14), resulting in an intraclass correlation coefficient (agreement) of 0.89.

The psychometric analysis of the AA-QLI-BRA showed the instrument’s adequate internal consistency, content validity and temporal stability, indicating its use in Brazil, allowing more precise quantification of the patients' demands perception, improving their care.

In this study, the mean DLQI scores indicated a moderate impact on QoL, although there is still no categorization of the AA-QLI scores regarding the dimension of impact on QoL. In general, generic questionnaires do not detect all the aspects affected in the patients’ lives, confirming the preference for specific instruments.^10^ The greater internal consistency and the centralized positioning of the AA-QLI-BRA items in the network confirm the superior psychometric performance of the DLQI in AA.^11^

Some items of the AA-QLI-BRA showed a psychometric behavior less aligned with the others and should be carefully evaluated during the instrument use. Item O21 is not directly linked to the clinical course of the disease. Item S6 may not be relevant in those who already have total/universal AA, item S4 may not be realistic in patients who are responding to treatment and item R15 may not reflect the main stigma of extensive AA: the impression of being submitted to chemotherapy.

Further research should explore the correlation between AA-QLI scores with clinical and cultural variables and affective disorder scores since AA can inflict a chronic contingency that favors the development of psychiatric illness.^12^, ^13^

This study has limitations related to the sampling of more severe patients with a higher level of schooling than the average of the Brazilian population.

In conclusion, the Brazilian version of the AA-QLI was adapted and proved to be valid and consistent, establishing itself as a useful instrument in clinical practice, as well as in clinical studies in AA.

## Financial support

None declared.

## Authors' contributions

Paula Rosa Coutinho Goulart Borges Mariottoni: Design and planning of the study; collection of data; drafting and editing of the manuscript; critical review of the literature; critical review of the manuscript; approval of the final version of the manuscript.

Leonardo Spagnol Abraham: Critical review of the literature; critical review of the manuscript; approval of the final version of the manuscript.

Leopoldo Duailibe Nogueira Santos: Critical review of the literature; critical review of the manuscript; approval of the final version of the manuscript.

Daniel Fernandes Melo: Critical review of the literature; critical review of the manuscript; approval of the final version of the manuscript.

Rodrigo Pirmez: Critical review of the literature; critical review of the manuscript; approval of the final version of the manuscript.

Paulo Müller Ramos: Design and planning of the study; drafting and editing of the manuscript; critical review of the literature; critical review of the manuscript; approval of the final version of the manuscript.

Hélio Amante Miot: Design and planning of the study; analysis and interpretation of data; statistical analysis; drafting and editing of the manuscript; critical review of the literature; critical review of the manuscript; approval of the final version of the manuscript.

## Conflicts of interest

None declared.
